# Evoking the Withdrawal Reflex via Successive Needle-Pricking on the Plantar and Dorsal Aspect of the Foot Increases the FMA of the Lower Limb for Poststroke Patients in Brunnstrom Stage III: A Preliminary Study

**DOI:** 10.1155/2020/3805628

**Published:** 2020-08-31

**Authors:** Cui-Cui Shen, Kuok-Tong Lei, Jin-Feng Jiang, Dan Miao, Jia-Wei Xiong

**Affiliations:** ^1^Department of Acupuncture and Moxibustion, The Affiliated Wuxi No. 2 People's Hospital of Nanjing Medical University, Wuxi 214000, Jiangsu Province, China; ^2^Key Laboratory of Acupuncture and Medicine Research of Ministry of Education, Nanjing University of Chinese Medicine, Nanjing 210023, Jiangsu Province, China; ^3^Department of Acupuncture and Moxibustion, Yizheng Hospital of Traditional Chinese Medicine, Yizheng 211400, Jiangsu Province, China; ^4^The Second Affiliated Hospital of Nanjing University of Chinese Medicine, Nanjing 210017, Jiangsu Province, China

## Abstract

The withdrawal reflex is a defensive reaction to nociceptive stimuli and can be used to regulate locomotor gait during rehabilitation. We investigated the effect of successive needle-pricking of the plantar and dorsal foot surfaces on poststroke lower limb function. Thirty-five hemiplegic patients, within one month after primary stroke, with an affected lower limb (Brunnstrom stage III) were randomly divided into intervention and control groups. Both groups received routine drug treatment, rehabilitation training, and upper limb acupuncture treatment on the hemiplegic side. The control group also received routine acupuncture on the hemiplegic side of the lower limb, while the intervention group received successive needle-pricking on the sole and instep of both the unaffected and affected side feet. Outcomes were assessed before inception (D0) and after three (D3) and six (D6) treatment days, using Brunnstrom stage (Ueda assessment), total Fugl–Meyer lower extremity assessment (FMA-LE) and its subscores (FMA-LE-ss), active lower limb range of motion (AROM-LL), Modified Ashworth Scale Score (MAS-LL), and manual muscle testing (MMT-LL). The Brunnstrom stage was better in the intervention group than in the control group at both D3 and D6 (*P* < 0.01). The total FMA-LE score and sections B, C, D, and G FMA-LE-ss were significantly better in the intervention group than in the control group at D3 and D6 (*P* < 0.05). The AROM-LL hip and knee flexion and hip extension improved more in the intervention group than in the control group (*P* < 0.05). In the intervention group, MAS-LL hip flexion significantly improved at D6 (*P* < 0.01). Improvement in lower limb joints on the MMT-LL in the intervention group exceeded that in the control group at D6 (*P* < 0.01). Successive needle-pricking on the plantar and dorsal foot aspects of Brunnstrom stage III in poststroke patients contributed to rapid lower limb motor function improvement via the withdrawal reflex. This trial is registered with ChiCTR1900020633.

## 1. Introduction

Stroke is a common cerebrovascular disease, characterised by high disability and mortality; it threatens the health of patients and increases the social burden [1]. Motor dysfunction of the lower limbs is one of the clinical symptoms of stroke and is often manifested by abnormal muscular tone of the affected lower limb, decreased muscle strength, and restricted joint movement [2]. According to the Brunnstrom theory, Brunnstrom stage III manifests as a lower limb extensor spasm; the hip, knee, and ankle joints of the affected lower limb are rigid and extended, resulting in a “hemiparetic gait” that requires lifting the hip joint and moving the leg in a circular motion when walking, which markedly affects walking ability and quality of life.

The withdrawal reflex was first described in 1910 by Sir Sherrington [3]. This reflex protects the body from injury in response to a nociceptive stimulus. The withdrawal reflex activates flexion of the limb and inhibits extension of the bilateral limbs when noxious cutaneous stimuli are present. This reflex also evokes the extensors and inhibits the flexors of the contralateral limb, in what is termed the crossed-extension reflex [4]. In addition to protective mechanisms, the nociceptive withdrawal reflex might have potential applications in the regulation of locomotion [5]. The ultimate withdrawal reflex is determined by the location of the evoking stimulus, which is within the cutaneous reflex receptive field (RRF) [6–8]. Repetitive electrical stimulation delivered at the arch of the foot and mid-forefoot of the lower limb has demonstrated the largest reflex responses [9].

Acupuncture has been used to treat various functional impairments after stroke for more than 2000 years. However, a meta-analysis has shown that acupuncture involving placement of needles into the conventional acupoints has no additional effect on motor recovery after stroke [10]. It has not yet been reported how lower limb motor dysfunction responds to successive manual needle-pricking on the plantar and dorsal aspect of the foot in poststroke patients in Brunnstrom stage III.

Thus, in this preliminary study, we designed procedures involving needle-pricking on the sole and instep of the foot to investigate its short-term effects on motor function recovery (based on lower limb Fugl–Meyer assessment (FMA)) of poststroke patients in Brunnstrom stage III.

## 2. Methods

### 2.1. Recruitment of Patients

This study was conducted in accordance with the principles of the Declaration of Helsinki, and the study protocol was approved by the Ethics Committee of Jiangsu Provincial Second Chinese Medicine Hospital, the Second Affiliated Hospital of Nanjing University of Chinese Medicine (no. 2017w1202) and registered at http://www.chitr.org.cn (registration no. ChiCTR1900020633). Patients signed an informed consent form when they enrolled in the study. All patients data were obtained from the Department of Neurology and Rehabilitation, Jiangsu Province Hospital of Chinese Medicine. The recruitment date was from December 5, 2017, to February 28, 2019. Thirty-five hemiplegic patients volunteered to participate in the study and were randomly divided into an intervention group (18 cases) and a control group (17 cases).

### 2.2. Inclusion Criteria

The inclusion criteria were as follows: (1) patients with stroke of the ischaemic type; (2) patients with a clinical diagnosis (computed tomography or magnetic resonance imaging) of stroke with a measurable neurological deficit; (3) patients within one month of their first stroke; (4) patients in whom the affected lower limb met Brunnstrom stage III criteria; (5) patients without cognitive impairment who could cooperate with the doctor's instructions.

### 2.3. Exclusion Criteria

The exclusion criteria were as follows: (1) patients with unstable vital signs; (2) those with a history of dementia, depression, or Parkinson's disease; (3) those with central nervous system complications: hemianopsia, agnosia, apraxia, vertigo, and numbness of the affected lower limb; (4) those with peripheral nervous system disease of affected lower limb; (5) those who were unable to cooperate or to follow the practitioner's instructions.

### 2.4. Study Design

**Figure 1:**
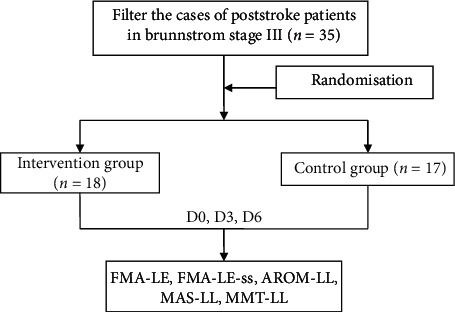
Flow diagram of the study.

Individual random numbers (*n* = 60) were generated by the random number table method to assign random numbers to 35 patients who met the inclusion criteria; the extracted numbers were not reused. Those with odd numbers were assigned to the control group (17 cases), and those with even numbers were assigned to the intervention group (18 cases). Patients' outcomes were assessed by a physical therapist at inception (D0) and after three days (D3) and after six days (D6) of treatment (Figure 1).

### 2.5. Intervention

All patients received routine drug treatment and rehabilitation physical training according to each patient's specific conditions. Acupuncture needles (0.30*∗*45 mm; Hwato, Suzhou New District, P. R. China) were used in this study. Acupuncture was performed by licensed acupuncturists. The control group received acupuncture on the upper limb (LI15, LI11, LI10, SJ5, LI4) and the lower limb (GB30, GB34, ST36, ST41, BL60) on the hemiplegic side. The needles were left in place for 30 minutes. The intervention group firstly received successive needle-pricking on the sole or instep and then received acupuncture on the upper limb of the hemiplegic side (LI15, LI11, LI10, SJ5, LI4) for 30 minutes. Both groups received acupuncture therapy once a day for six days.

**Figure 2:**
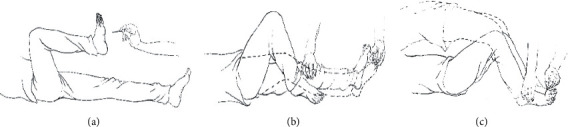
(a) Repeated needle-pricking on the arch of the affected foot evokes the withdrawal reflex. (b) Repeated needle-pricking on the arch and restriction of the knee of the unaffected limb evokes the crossed-extension reflex of the affected limb. (c) Keeping both knees flexed and restricting movement of the ankles, repeated needle-pricking on the instep evokes hip lifting. The dotted line represents the direction of the evoked movement.

The specific operations of successive needle-pricking on the sole or instep were as follows (Figure 2).

First, the patient was placed in a supine position with the legs straightened naturally. The arch of the affected foot was pricked continuously until the withdrawal reflex was evoked. At the end of the reflexive motion, needle-pricking was performed again. Successive needle-pricking did not terminate until the maximum range of lower extremity motion was achieved. It usually required about 3–7 repetitive needle pricks to produce continuous knee and hip flexion until the maximum range of motion. This intervention was executed three times per procedure (Figure 2(a)).

Second, the patient was placed in a supine position with the affected knee joint flexed while the unaffected knee was extended and immobilised during the intervention. The arch of the unaffected foot was pricked continuously until the affected lower limb extended to its maximum range. This intervention was executed three times per procedure (Figure 2(b)).

Third, the patient was placed in a supine position with both knees flexed, while the knee and ankle joints of both legs were kept immobilised during the treatment. The insteps of both feet were then pricked repeatedly. The immobilised joints forced the patient to perform bridge lifts with their hips owing to the needle-pricking. This bridge posture had to be sustained for more than 10 seconds, and the entire process was repeated three times (Figure 2(c)).

### 2.6. Outcome Measures

The primary outcomes of the study were the Brunnstrom stage (based on the Ueda assessment), FMA of the lower extremity (FMA-LE), and subscores of the FMA-LL (FMA-LE-ss). The secondary outcomes were active range of motion of the lower limb (AROM-LL), the Modified Ashworth Scale Score for the lower limb (MAS-LL), and the manual muscle test score for the lower limb (MMT-LL).

### 2.7. Statistical Analyses

Data analyses were performed using SPSS v.22.0 (SPSS, Inc., Chicago, IL, USA). Two-way repeated-measures analysis of variance was used to compare the FMA-LE, FMA-LE-ss, and AROM-LL. The Mann–Whitney *U* test was applied to evaluate the Brunnstrom stage (Ueda assessment), MAS-LL, and MMT-LL. A *P* value < 0.05 was considered statistically significant.

## 3. Results

### 3.1. Patient Characteristics

The baseline characteristics, such as age and course of disease, were similar between the intervention and control groups (*P* > 0.05; Table 1).

### 3.2. Primary Outcomes of the Two Groups

The Mann–Whitney *U* test showed that Brunnstrom stage in the intervention group was better than that in the control group at both D3 and D6 (*P* < 0.01; Table 2).

The total FMA-LE score in the intervention group increased from 10.94 ± 2.87 to 16.33 ± 4.79 at D3 (*P* < 0.01) and further increased to 19.89 ± 5.16 at D6 (*P* < 0.01). The total FMA-LE score in the intervention group was significantly higher than that in the control group at D3 and D6 (*P* < 0.01; Table 3).

**Figure 3:**
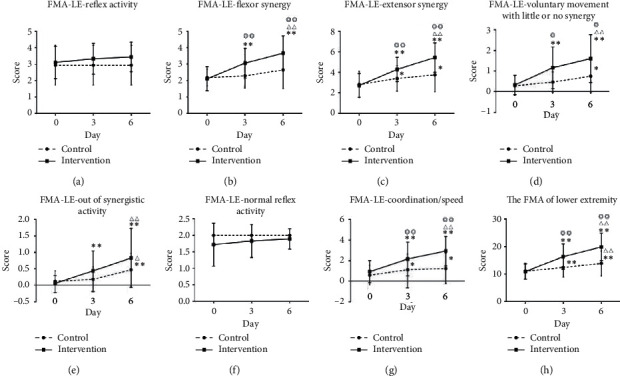
(a–g) The comparison of the FMA-LE-ss between the control group and the intervention group at D3 and D6. (h) The comparison of the total FMA between the control group and the intervention group at D3 and D6. ^*∗*/*∗∗*^D0 compared with D3 or D6; ^Δ/ΔΔ^D3 compared with D6; ^◎/◎◎^between-group comparison (*P* < 0.05/*P* < 0.01).

In terms of FMA-LE-ss, in the intervention group, scores for sections B, C, D, E, and G of the FMA-LE were significantly improved at D3 and D6 compared with those at the baseline (*P* < 0.01). Sections B, C, D, and G of the FMA-LE-ss in the intervention group were markedly higher at both D3 and D6 than those in the control group (*P* < 0.05; Table 3, Figure 3).

### 3.3. Secondary Outcomes of the Two Groups

**Figure 4:**
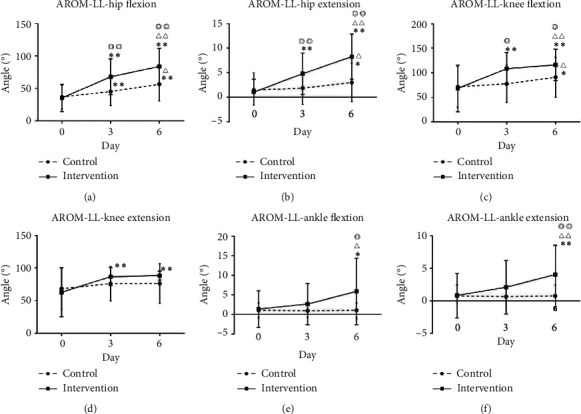
The hip flexion, knee extension, and ankle flexion/extension angles were measured in the supine position. For hip extension and knee flexion, the contralateral position was used. (a–f) The comparison of AROM between the control group and the intervention group at D3 and D6. ^*∗*/*∗∗*^D0 compared with D3 or D6;^Δ/ΔΔ^D3 compared with D6; ^◎/◎◎^between-group comparison (*P* < 0.05/*P* < 0.01).

For the AROM-LL, the flexion and extension of the hip and knee improved significantly over time (D3 and D6) (*P* < 0.01) compared with those at baseline in the intervention group, while the flexion of the hip and knee and the extension of the hip improved markedly in the intervention compared with those in the control group (*P* < 0.05). The flexion and extension of the ankle in the intervention group improved at D6 compared with those in the control group (*P* < 0.05; Table 4, Figure 4).

For the MAS-LL, only hip flexion significantly improved in the intervention group compared with the control group after six days of treatment (*P* < 0.01; Table 5).

In the MMT-LL, hip flexion, knee extension, and ankle flexion/extension improved in the intervention group compared with the control group at D6 (*P* < 0.05), while hip extension and knee flexion significantly improved in the intervention group compared with the control group at both D3 and D6 (*P* < 0.01; Table 6).

### 3.4. Adverse Events

In terms of adverse effects, four patients had petechiae related to the needle-pricking process.

## 4. Discussion

As conventional acupuncture, which has been widely used in the rehabilitation of hemiplegic stroke patients, has no more than a placebo effect on motor recovery in stroke rehabilitation [10, 11], we here investigated the effect of needle-pricking on motor recovery. In our study, we designed three intervention methods that combined the theory of the withdrawal reflex and successive needle-pricking. The first intervention induced the hip, knee, and ankle of the lower limbs to achieve maximum flexion; the second intervention aimed to induce the crossed-extension reflex, while the third intervention strengthened bridging activity to improve the muscles involved in trunk stability. The control group and the intervention group were compared at baseline and no difference in any measure was found. We provide evidence that the withdrawal reflex, evoked by successive needle-pricking on the plantar and dorsal aspect of the foot in the intervention group, resulted in significant improvement of motor function of the affected lower limb in poststroke patients in Brunnstrom stage III.

Acupuncture integrates the stimulation signals through the primary movement centre of the spinal cord and transmits them to the paralysed muscles, exciting the muscles and preventing atrophy. Repetitive acupuncture can excite the advanced motor centre of the cerebral cortex, restore and reshape the normal nerve reflex pathway, regulate the concentration and distribution of neurotransmitters, restore the physiological balance in the brain, and promote the compensation and reconstruction of the affected side by the uninvolved brain tissue [12–14]. According to neurophysiology, when a sensory stimulus, such as needle-pricking, pain, or burning is encountered at the lower limb extremity, the stimulus signal is transmitted to the spinal cord through the peripheral sensory nerves, causing the withdrawal reflex on the stimulated side to contract the muscle fibres of the lower limb. At the same time, the stimulation signal is transmitted to the contralateral motor nerve at the level of the spinal cord, causing extension of the contralateral limb [15]. Bridge exercises are often used to strengthen trunk stability muscles [16]. Repetitive needle-pricking can enhance the performance of bridge exercises, which facilitates control of the hip and knee and helps in training of the trunk muscles, contributing to the breaking of the synergy pattern and establishing separation of limb movement. Our design attempted to induce the flexor reflex to antagonise the dominant motion mode of the lower extremity extensor to attain the subjective ability of the correct motion mode. The treatment did not aim to strengthen the patient's flexor or extensor movement pattern during walking.

A previous study using cutaneous electrical stimulation suggested that maximal flexion could be evoked by stimulation at the arch of the foot [17]. In our first and second interventions, needle-pricking was repeated at the end of the reflexive motion to induce the maximum flexion reflex. The total score of the FMA-LE in the intervention group was significantly greater than that in the control group both at D3 and D6 (*P* < 0.01). The scores of sections B, C, D, and G of the FMA-LE-ss in the intervention group were significantly higher than those in the control group at both D3 and D6 (*P* < 0.05). After six days' treatment, the patients' FMA-LE scores in the intervention group increased from 10.94 ± 2.87 to 19.89 ± 5.16, while those in the control group increased from 11.06 ± 2.90 to 13.88 ± 4.72. In contrast, it required 28 days of conventional acupuncture treatment to increase the FMA-LL from 14.11 ± 4.59 to 20.73 ± 4.780 [18]. Another study showed that, after 30 days' treatment, the FMA-LL increased from 7.37 ± 3.53 to 19.27 ± 8.11 in the exercise therapy group, while it improved from 7.25 ± 3.55 to 24.56 ± 7.82 in the group that received both exercise therapy and electroacupuncture [19]. Our findings provide clinical evidence that successive needle-pricking on the arch of the foot significantly improved the lower limb motor function of poststroke patients and was clinically more effective.

After six days of intervention treatment, besides the total score of FMA-LE, sections B, C, D, E, and G of the FMA-LE-ss and the flexion and extension of the hip and knee in AROM-LL improved at D3 and were even better at D6 (*P* < 0.05). At D6, the flexion and extension of the ankle also showed a significant difference (*P* < 0.05). The Brunnstrom stage in the intervention group had advanced at D3 (*P* < 0.01). Compared with those in the control group, the total score of FMA-LE, sections B, C, D, and G of the FMA-LE-ss, the flexion and extension of the hip, and the flexion of the knee in the AROM-LL were higher both at D3 and D6 in the intervention group (*P* < 0.05). The flexion and extension of the ankle in AROM-LL were significantly more improved in the intervention group than in the control group at D6 (*P* < 0.05). The data showed the rapid onset of improvement in the lower limb hemiplegia after repetitive needle-pricking. After the treatment, the patient subjectively felt that the stride length of the affected side was increased, the support strength was increased, and the support time was prolonged. The withdrawal reflex induced by successive needle-pricking activated cocontraction of multiple muscles and maintained the dynamic continuous flexion of the lower limbs to antagonise the extensor synergy of the affected lower limbs, which eventually broke the synergy pattern and accelerated advances in improvement. The onset period of our design was shorter than that of cocontraction of multiple muscles achieved by functional electrical stimulation [20]. In this way, successive needle-pricking appears to promote the recovery of lower extremity motor function in a shorter time.

For the MAS, hip flexion was significantly improved in the intervention group than that in the control group after six days of treatment (*P* < 0.01). The improvement of hip flexion is beneficial for patients, as it alleviates extensor spasms and prevents venous thrombosis. The strength of the lower limb muscles has an important impact on the balance and walking function in patients with poststroke hemiplegia [21]. The significant improvement in hip flexion/extension, knee flexion/extension, and ankle flexion/extension in the MMT-LL in the intervention group indicated the recovery of muscle strength, which would enhance the quality of life of the patients. Hip extension and knee flexion significantly improved in the intervention group compared with the control group at D3 (*P* < 0.01), which conforms to the classic neurodevelopmental theory in that the recovery speed of proximal joints is faster than that of the distal joints. However, manual measurement may result in some error; thus, in further clinical trials, indicators such as electromyography should be added.

Safety is a basic principle for clinical therapeutic methods. Acupuncture is a traditional Chinese medicine practice in which thin needles are inserted into the body. It addresses the energy (Qi) in the meridian, but from a neurophysiological perspective, it also acts as mechanical stimulation. Acupuncture has been used for poststroke paralysis. Mounting evidence has demonstrated the safety of acupuncture. In this study, needle-pricking involved inserting the tip of the needle to no more than the subcutaneous level. After the intervention, the acupuncturist strictly sanitised the corresponding irritated part. Patients may occasionally experience petechiae during the course of treatment but do not exhibit more marked adverse events, such as hematoma and numbness caused by effects on the peripheral nerves. Hence, acupuncture's safety is guaranteed.

Our study had some limitations. The sample size was small (35 cases). The study period was relatively short and did not include long-term follow-up observations. There was a degree of subjectivity in the measurements conducted by the evaluator. Conventional rehabilitation treatment may have created a certain bias in this study. However, these factors do not affect the rapidity of recovery and the changes in the FMA score of poststroke patients.

In conclusion, successive needle-pricking on the plantar and dorsal aspects of the foot is effective for promoting muscle strength, balance ability, and voluntary movement in patients with poststroke hemiplegia. The results of this study warrant further implementation of this safe and convenient treatment in the clinic.

## Figures and Tables

**Table 1:** Patient's baseline characteristics.

Group	Patient	Male	Female	Age (years)	*P* value	Days from stroke	*P* value
Intervention group	18	14	4	63.83 ± 9.23	*P*=0.354	9.28 ± 5.45	0.185
Control group	17	11	6	66.53 ± 7.60		7.29 ± 5.09	

**Table 2:** Comparison of the Brunnstrom stage (Ueda assessment) of the lower limb between the two study groups at D3 and D6.

Treatment time (day)	U value	*P* value
After 3 days (D3)	66.50	0.003^*∗∗*^
After 6 days (D6)	46.00	≤0.001^*∗∗*^

^*∗∗*^ *P* < 0.01: between-group comparisons.

**Table 3:** Fugl–Meyer assessment of the lower extremity (FMA-LE and FMA-LE-ss).

Group	FMA (max score)	Result (score)			*P* value (within group)		
		Baseline (D0)	After 3 days (D3)	After 6 days (D6)	*P*1	*P*2	*P*3
Intervention group	Section A: reflex activity (4)	3.11 ± 1.02	3.33 ± 0.97	3.44 ± 0.92	0.078	0.093	0.057
	Section B: flexor synergy (6)	2.11 ± 0.75	3.06 ± 0.93^*∗∗*^	3.67 ± 1.08^*∗∗*^	0.001^##^	≤0.001^##^	0.001^##^
	Section C: extensor synergy (8)	2.72 ± 1.17	4.28 ± 1.22^*∗∗*^	5.44 ± 1.46^*∗∗*^	≤0.001^##^	≤0.001^##^	≤0.001^##^
	Section D: voluntary movement with little or no synergy (4)	0.33 ± 0.48	1.17 ± 1.043^*∗*^	1.61 ± 1.19^*∗*^	≤0.001^##^	≤0.001^##^	0.002^##^
	Section E: out of synergy activity (4)	0.06 ± 0.23	0.44 ± 0.61	0.83 ± 0.92	0.008^##^	≤0.001^##^	0.001^##^
	Section F: normal reflex activity (2)	1.72 ± 0.66	1.83 ± 0.51	1.89 ± 0.32	0.166	0.083	0.399
	Section G: coordination/speed (6)	0.94 ± 1.10	2.17 ± 1.68^*∗∗*^	2.94 ± 1.47^*∗∗*^	≤0.001^##^	≤0.001^##^	0.001^##^
	Sections A‒G: total score (32)	10.94 ± 2.87	16.33 ± 4.79^*∗∗*^	19.89 ± 5.16^*∗∗*^	≤0.001^##^	≤0.001^##^	≤0.001^##^

Control group	Section A: reflex activity (4)	2.94 ± 1.24	2.94 ± 1.24	2.94 ± 1.24	1.000	1.000	1.000
	Section B: flexor synergy (6)	2.18 ± 0.52	2.29 ± 0.77	2.65 ± 1.16	0.332	0.056	0.055
	Section C: extensor synergy (8)	2.82 ± 1.33	3.41 ± 1.27	3.76 ± 1.71	0.013^#^	0.021^#^	0.208
	Section D: voluntary movement with little or no synergy (4)	0.29 ± 0.47	0.47 ± 0.51	0.76 ± 0.83	0.083	0.041^#^	0.096
	Section E: out of synergy activity (4)	0.12 ± 0.33	0.18 ± 0.39	0.47 ± 0.51	0.336	0.009^##^	0.020^#^
	Section F: normal reflex activity (2)	2.00 ± 0.00	2.00 ± 0.00	2.00 ± 0.00	1.000	1.000	1.000
	Section G: coordination/speed (6)	0.59 ± 1.04	1.12 ± 1.57	1.24 ± 1.52	0.034^#^	0.017^#^	0.431
	Sections A‒G: total score (32)	11.06 ± 2.90	12.41 ± 3.64	13.88 ± 4.72	0.016^##^	0.004^##^	0.003^##^

*P*1: D0 compared with D3, *P*2: D0 compared with D6, *P*3: D3 compared with D6. ^#^*P* < 0.05, ^##^*P* < 0.01: within-group comparisons ^*∗*^*P* < 0.05, ^*∗∗*^*P* < 0.01: between-group comparisons.

**Table 4:** Active range of motion of the lower limb (AROM-LL).

Group	AROM-LL	Results (°)			*P* value (within group)		
		Baseline (D0)	After 3 days (D3)	After 6 days (D6)	*P*1	*P*2	*P*3
Intervention group	Hip flexion	35.47 ± 21.68	67.83 ± 28.59^*∗∗*^	83.80 ± 28.73^*∗∗*^	≤0.001^##^	≤0.001^##^	0.001^##^
	Hip extension	1.05 ± 2.66	4.81 ± 4.35^*∗∗*^	8.27 ± 4.73^*∗∗*^	≤0.001^##^	≤0.001^##^	≤0.001^##^
	Knee flexion	68.38 ± 48.88	108.44 ± 33.68^*∗*^	116.16 ± 33.24^*∗*^	≤0.001^##^	≤0.001^##^	≤0.001^##^
	Knee extension	62.77 ± 38.47	86.66 ± 14.14	88.33 ± 7.07	0.004^##^	0.005^##^	0.638
	Ankle flexion	1.38 ± 4.79	2.66 ± 5.45	5.88 ± 8.72^*∗*^	0.133	0.017^#^	0.027^#^
	Ankle extension	0.83 ± 3.53	2.11 ± 4.22	4.05 ± 4.62^*∗∗*^	0.133	0.003^##^	0.009^##^

Control group	Hip flexion	36.88 ± 18.81	45.17 ± 21.89	56.52 ± 26.42	0.006^##^	≤0.001^##^	0.030^#^
	Hip extension	1.47 ± 3.59	1.88 ± 3.46	3.00 ± 4.01	0.248	0.041^#^	0.035^#^
	Knee flexion	71.58 ± 43.00	77.82 ± 39.11	90.88 ± 41.72	0.344	0.026^#^	0.013^#^
	Knee extension	68.41 ± 32.23	75.88 ± 27.34	76.17 ± 31.00	0.157	0.264	0.940
	Ankle flexion	1.05 ± 2.01	0.94 ± 1.81	1.05 ± 2.01	0.743	1.000	0.332
	Ankle extension	0.76 ± 1.75	0.64 ± 1.49	0.76 ± 1.75	0.745	1.000	0.336

*P*1: D0 compared with D3, *P*2: D0 compared with D6, *P*3: D3 compared with D6. ^#^*P* < 0.05, ^##^*P* < 0.01: within-group comparisons ^*∗*^*P* < 0.05, ^*∗∗*^*P* < 0.01: between-group comparisons.

**Table 5:** Comparison of MAS-LL of the lower limb between the two study groups at D3 and D6.

Lower extremity joints	Treatment time (day)	*U* value	*P* value
Hip flexion	After 3 days (D3)	111.50	0.173
	After 6 days (D6)	75.00	0.009^*∗∗*^

Hip extension	After 3 days (D3)	127.50	0.405
	After 6 days (D6)	128.50	0.424

Knee flexion	After 3 days (D3)	136.00	0.590
	After 6 days (D6)	136.50	0.590

Knee extension	After 3 days (D3)	153.00	1.000
	After 6 days (D6)	149.00	0.909

Ankle flexion	After 3 days (D3)	129.00	0.443
	After 6 days (D6)	107.00	0.134

Ankle extension	After 3 days (D3)	121.00	0.303
	After 6 days (D6)	105.00	0.118

^*∗∗*^ *P* < 0.01: between-group comparisons.

**Table 6:** Comparison of MMT-LL of the lower limb between the two study groups at D3 and D6.

Lower extremity joints	Treatment time (Day)	*U* value	*P* value
Hip flexion	After 3 days (D3)	96.50	0.062
	After 6 days (D6)	75.00	0.009^*∗*^

Hip extension	After 3 days (D3)	61.00	0.002^*∗∗*^
	After 6 days (D6)	52.00	0.001^*∗∗*^

Knee flexion	After 3 days (D3)	70.50	0.005^*∗∗*^
	After 6 days (D6)	51.50	≤0.001^*∗∗*^

Knee extension	After 3 days (D3)	110.50	0.163
	After 6 days (D6)	73.50	0.007^*∗*^

Ankle flexion	After 3 days (D3)	110.50	0.163
	After 6 days (D6)	73.50	0.007^*∗*^

Ankle extension	After 3 days (D3)	137.00	0.613
	After 6 days (D6)	83.00	0.020^*∗*^

^*∗*^ *P* < 0.05, ^*∗∗*^*P* < 0.01: between-group comparisons.

## Data Availability

The data used in this study are available upon reasonable request from the corresponding author.
